# Characterization of the Structure and Physicochemical Properties of Soluble Dietary Fiber from Peanut Shells Prepared by Pulsed Electric Fields with Three-Phase Partitioning

**DOI:** 10.3390/molecules29071603

**Published:** 2024-04-03

**Authors:** Rui Fan, Lei Wang, Huihui Cao, Ruihuan Du, Shuo Yang, Yanhua Yan, Baiqin Zheng

**Affiliations:** 1 Department of Nutrition and Food Hygiene, School of Public Health, Peking University, Beijing 100191, China; fanruirf@bjmu.edu.cn; 2 Tangshan Food and Drug Comprehensive Testing Center, Tangshan 063000, China; wanglei3730217@163.com (L.W.); hhcao66@126.com (H.C.); dudu12345@126.com (R.D.); yangyangs@126.com (S.Y.); 3 Key Laboratory of Quality Evaluation and Nutrition Health of Agro-Products, Ministry of Agriculture and Rural Affairs, Tangshan 063000, China; 4 Hebei Agricultural Products Quality and Safety Testing Innovation Center, Tangshan 063000, China

**Keywords:** peanut shell, soluble dietary fiber, physicochemical, functional properties, pulsed electric fields, three-phase partitioning

## Abstract

This study evaluated the impact of pulsed electric fields (PEFs) combined with three-phase partitioning (TPP) extraction methods on the physicochemical properties, functional properties, and structural characterization of the soluble dietary fiber (SDF) derived from peanut shells (PS). The findings of this study indicated that the application of a PEF-TPP treatment leads to a notable improvement in both the extraction yield and purity of SDF. Consequently, the PEF-TPP treatment resulted in the formation of more intricate and permeable structures, a decrease in molecular weight, and an increase in thermal stability compared to SDFs without TPP treatment. An analysis revealed that the PEF-TPP method resulted in an increase in the levels of arabinose and galacturonic acid, leading to enhanced antioxidant capacities. Specifically, the IC_50_ values were lower in SDFs which underwent PEF-TPP (4.42 for DPPH and 5.07 mg/mL for ABTS) compared to those precipitated with 40% alcohol (5.54 mg/mL for DPPH, 5.56 mg/mL for ABTS) and PEF75 (6.60 mg/mL for DPPH, 7.61 mg/mL for ABTS), respectively. Notably, the SDFs which underwent PEF-TPP demonstrated the highest water- and oil-holding capacity, swelling capacity, emulsifying activity, emulsion stability, glucose adsorption, pancreatic lipase inhibition, cholesterol adsorption, nitric ion adsorption capacity, and the least gelation concentration. Based on the synthesis scores obtained through PCA (0.536 > −0.030 > −0.33), which indicated that SDFs which underwent PEF-TPP exhibited the highest level of quality, the findings indicate that PEF-TPP exhibits potential and promise as a method for preparing SDFs.

## 1. Introduction

China boasts the largest peanut production, consumption, and export trade, concurrently holding the highest cultivated area and annual output of peanuts globally [1]. Annually, the peanut industry generates a substantial volume of peanut hulls, which possess a significant nutritional value characterized by protein, crude fiber, hemicelluloses, and crude fat contents ranging from 4.8% to 7.2%, 65.7% to 79.3%, 10.1% to 11.6%, and 1.2% to 1.8%, respectively [2]. Furthermore, the peanut’s shell is rich in a diverse array of minerals including calcium, iron, copper, zinc, manganese, boron, aluminum, strontium, barium, sodium, phosphorus, potassium, and magnesium. Additionally, it contains beneficial medicinal compounds such as β-sitosterol, saponin, and xylose [3]. It was reported that peanut shells (PSs) possess a significant cellulose content that holds considerable economic worth [4]. Previous studies have primarily concentrated on the utilization of the PS as a material [5,6] and biomass source [7], neglecting its potential as a valuable dietary fiber. This oversight warrants further investigation. In contemporary times, there has been an insufficient amount of focus on the by-products derived from the processing of peanut products, specifically PSs, which are either discarded or utilized solely as animal feed. This limited utilization of the active ingredients within PSs leads to an escalation in biological resource wastage, environmental pollution, and processing expenses. Hence, the environmentally sustainable utilization of PSs has the potential to mitigate the wastage of biological resources and enhance the economic revenue of fruit farmers.

Dietary fiber (DF) encompasses a diverse range of compounds, such as plant oligosaccharides and polysaccharides (e.g., cellulose, hemicellulose, pectin, gums, resistant starch, lignin), as well as non-carbohydrate components. These constituents have been found to exert advantageous physiological effects on human health [8]. In recent years, numerous health benefits linked to the consumption of DF have been extensively documented, encompassing a decreased susceptibility to heart disease, diabetes, obesity, and select types of cancer [9]. The classification of DF into insoluble dietary fiber (IDF) and soluble dietary fiber (SDF) is determined by its water solubility. SDF, in contrast to IDF, has the ability to undergo complete hydrolysis in the colon, thereby facilitating the growth of intestinal probiotics, inhibiting lipid transport and cardiovascular disease, as well as scavenging free radicals [10]. Numerous studies have demonstrated the potential of utilizing SDF derived from material by-products as a viable fat substitute [11].

Presently, the food production industry utilizes diverse extraction techniques for dietary fiber, including chemical, biological, and physical approaches, with several methods being employed to augment the functionality of SDF [12]. In contrast to the chemical method involving a chemical reagent residue, physical extraction, as an emerging technology, has been applied in an extensive amount of applications in extracting nutritional/functional compounds. This technique effectively increases the proportion of SDF within the overall DF content. The application of pulsed electric field (PEF) technology involves the administration of brief, high-voltage electrical pulses to a given product. Upon exposure to an electric field, a living cell experiences the generation of an electrostatic charge, resulting in the development of an electrical field potential. Once this electrical potential surpasses a critical threshold, the weak regions of the cell membrane undergo reversible or irreversible electroporation, thereby facilitating the liberation of cytoplasmic fluid and cellular constituents [13,14]. Our team has documented the early-stage achievement of high efficiency in the preparation of SDF from PSs through the use of a pulsed electric field (PEF) [15], while another team prepared SDF from PSs using microwave technology [16]. Furthermore, the properties of these SDFs exhibit notable superiority when compared to both the conventional method and the enzyme solution method. However, the preceding investigation revealed the consistent presence of a technical bottleneck during the purification process of dietary fiber. Currently, there is a lack of literature regarding the purification method of SDF from PSs. The crude dietary fiber obtained from PSs has a low purity of approximately 56% [16], potentially containing proteins and starch polysaccharides. The purification process for the application of SDF remains underexplored in academic research. The method of three-phase partitioning (TPP) is a recently developed non-chromatographic technique utilized for the separation and extraction of small organic compounds from protein lipids and oils. This technique employs salting out isoelectric precipitation and cosolvent precipitation in solutions containing tert-butanol and ammonium sulfate [17]. TPP exhibits notable advantages such as its high extraction efficiency, low energy consumption, and its ability to preserve the physiological activity of the original materials.

Currently, there is limited research examining the structural and functional characteristics of SDF derived from peanut shells. In this study, the authors employed PEF in conjunction with TPP to extract SDF from peanut shells. Subsequently, they conducted an analysis to investigate the impact of this extraction method on the structure, physicochemical properties, and functional activity of the obtained SDF. The outcomes of this research endeavor may serve as a theoretical foundation for future investigations concerning SDF derived from peanut shells and the development of associated products.

## 2. Results and Discussion

### 2.1. Proximate Composition Analysis

Table 1 presents a comprehensive overview of the basic components of PSs and SDF. Notably, the total dietary fiber content in PSs was found to be 83.91 ± 0.75 g/100 g, surpassing the levels observed in pear residue (56.90 g/100 g) [18] and rice bran (27.04 g/100 g) [19]. This observation underscores the notion that the PS serves as a highly favorable source of dietary fiber.

Following various treatments, the SDFs exhibited a greater overall sugar content compared to the PS. Notably, the application of the PEF treatment demonstrated a more pronounced impact on enhancing the total sugar content. The findings indicate a significant increase in total sugar content subsequent to PEF modification, potentially attributable to the conversion of insoluble fibers into soluble polysaccharides. These outcomes align with prior research outcomes [20,21].

In comparison to PSs, both SDFs exhibited diminished levels of impurities, including crude protein, crude fat, and ash. Notably, PEF-TPP SDF demonstrated a significant reduction of 85.2% in its crude protein content, suggesting the efficacy of three-phase partitioning in eliminating protein impurities. This indicated that the use of a TPP treatment could increase the purity of SDF.

### 2.2. Physicochemical Properties

#### 2.2.1. WHC, SC, and OHC

SDF is a mixture of complex carbohydrates with a polysaccharide structure. The favorable physiological functions of SDF stem from its notable physical properties, including its effective water retention, viscosity, binding capabilities, and adsorption potential. Its hydraulic and expansive force serves as a significant metric for assessing the quality of the SDF, with higher values generally indicating superior adsorption performance [22]. This can be attributed to the abundance of hydrophilic groups present in SDF, which facilitate the binding of water molecules and the subsequent expansion, resulting in volumetric effects. Consequently, SDF aids in enhancing satiety, reducing food intake, and mitigating obesity [23]. Its water-holding capacity (WHC), swelling capacity (SC), and oil-holding capacity (OHC) are significant parameters for assessing the quality and physiological functionality of SDF.

The WHC refers to the material’s ability to retain water, thereby mitigating the dehydration and shrinkage of the product, while also facilitating digestion within the human body [24]. As demonstrated in Table 2, the WHC of PEF-TPP SDF (5.67 ± 0.67 g/g) significantly surpassed that of PEF75 (4.96 ± 0.17 g/g) (*p* < 0.05) and PEF40 (3.98 ± 0.29 g/g) (*p* < 0.05), attributable to the heightened porosity of PEF-TPP SDF and the increased availability of hydrogen bond and water-binding sites. Furthermore, it is important to note that the WHC of SDF is influenced by various factors, including the processing conditions and charge density [15]. The WHC of PEF-TPP SDF was found to be higher than that of wheat bran (2.18 ± 0.18 g/g) [25], papaya peel (5.26 ± 0.15 g/g) [26], and orange peel (3.63 ± 0.21 g/g) [27]. This finding highlights the potential of the PS as a valuable source of dietary fiber, with the added benefit of an improved WHC when treated with PEF-TPP.

In contrast to the WHC, the SC is influenced by various factors including the network density, molecular size, and the cellulose components of the fiber [24]. According to Table 2, the SC values of the PEF-TPP SDF (6.96 ± 0.88 mL/g) and the PEF75 SDF (5.49 ± 0.27 mL/g) were significantly higher (*p* < 0.05) compared to that of the PEF40 SDF (4.57 ± 0.36 mL/g). The water-related properties of dietary fiber, including the WHC and SC, were influenced by the specific components and physical characteristics (e.g., porosity and crystallinity) of the fiber. It is hypothesized that the increased WHC and SC observed in the dietary fiber treated with PEF-TPP may be attributable to an increase in the proportion of short-chain dietary fiber and its surface area [28].

The inclusion of a high OHC in the SDF proved advantageous in maintaining the stability of high-fat foods. The OHC levels of the PEF-TPP SDF (3.89 ± 0.41 g/g) and the PEF75 SDF (3.74 ± 0.14 g/g) exhibited a significant increase (*p* < 0.05) compared to those of the PEF40 SDF (2.88 ± 0.20 g/g). The SDF obtained with PEF-TPP had highest OHC values, which may be related to their better complex porous structure and surface area. Furthermore, the OHC levels of all the samples surpassed those of the SDF derived from pear pomace (2.77 g/g) [29] and rice hull (1.85 ± 0.15 g/g) [30]. Prior research has demonstrated that fiber particles possess the ability to absorb and amalgamate oily constituents [31], thereby potentially enhancing the palatability of food [32]. Consequently, SDFs after PEF-TPP treatment hold promise as valuable resources for the functional food sector.

#### 2.2.2. EA, ES, and LGC

Their emulsifying properties, specifically the emulsifying activity (EA), emulsion stability (ES), and least gelation concentration (LGC), are crucial factors in promoting the solubilization or dispersion of substances and improving their resistance to emulsion rupture [11].

The EA property of the SDF was evidenced by its interfacial absorption, characterized by a high tensile strength that promoted the development of compact films. These films, enveloping lipid droplets, efficiently hindered coalescence by establishing a hydrophilic barrier at the interface of the oil and water phases [8].

As demonstrated in Table 2, the EA values for the PEF40, PEF75, and PEF-TPP were 66.37 ± 1.83 mL/100 mL, 73.69 ± 1.01 mL/100 mL, and 79.69 ± 2.36 mL/100 mL, respectively. Notably, the EA value for the PEF-TPP was significantly higher than those obtained with the other methods (*p* < 0.05). Furthermore, the PEF-TPP SDF and PEF75 SDF exhibited an obviously higher ES value compared to that of the PEF40 SDF (70.36 ± 2.13 mL/100 mL, 63.54 ± 1.24 mL/100 mL, and 55.11 ± 1.13 mL/100 mL). The stability of the emulsion is influenced, in part, by its composition, specifically the neutral sugar chains of the RG I region and its molecular mass [33,34]. The highest content of the RG I region in the SDF obtained with PEF-TPP may contribute to a higher emulsion stability. The molecular mass of the emulsion determines its conformation when in solution, affecting its ability to form a protective layer around oil droplets and ensure steric stabilization. Therefore, the superior emulsifying and stabilizing properties of PEF-TEE SDF may be attributable to its small molecular mass and large neutral sugar chains.

The gelation capacity, as assessed via the LGC, plays a critical role in determining the appropriateness of associated food products. Typically, a lower LGC value signifies a higher quality gelation property [35].

The LGC values obtained with PEF40, PEF75, and PEF-TPP were 11.26 ± 0.71% (*w*/*v*), 10.02 ± 0.31% (*w*/*v*), and 8.18 ± 0.28% (*w*/*v*), respectively. The smallest value of the LGC was clearly evident in the PEF-TPP SDF, as indicated by the statistical significance (*p* < 0.05). The findings indicate that the SDF generated through the application of PEF-TPP displayed a notable capacity for gelation.

Primarily, the treatment of TPP has the potential to notably enhance the WHC, OHC, SC, EA, and ES of SDF, while simultaneously reducing its LGC. Consequently, the PEF-TPP SDF exhibits superior processing characteristics, making it suitable for extensive utilization within the food industry.

### 2.3. Functional Properties

#### 2.3.1. Glucose Absorption Capacity (GAC)

**Figure 1:**
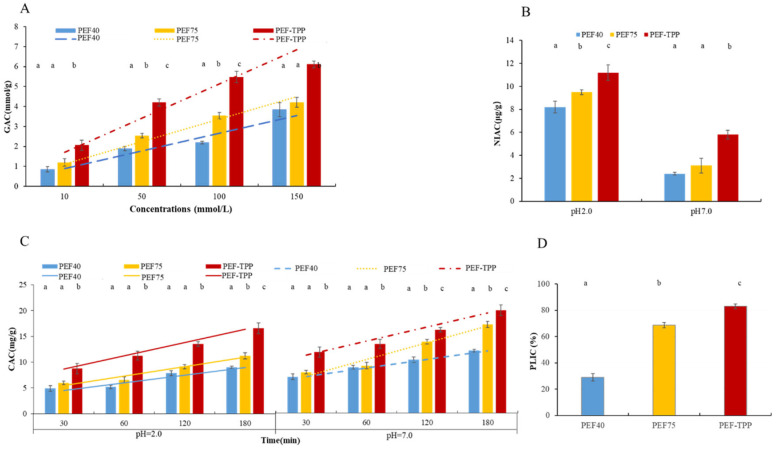
Functional properties of SDFs obtained with different extraction methods. Glucose absorption capacity—GAC (**A**); Nitrite ion adsorption capacity—NIAC (**B**); Cholesterol absorption capacity—CAC (**C**); and Pancreatic lipase inhibition capacity—PLIC (**D**). Different letters are statistically different (*p* < 0.05).

The mechanism of hypoglycemic action includes the capacity for glucose absorption. Figure 1A illustrates that SDFs obtained through various extraction methods exhibit effective glucose adsorption at different glucose concentrations. Among these methods, PEF-TPP demonstrates the highest GAC value within the range of 10–150 mmol/L. Additionally, PEF-TPP exhibits a significantly stronger GAC value compared to PEF75 and PEF40 at all glucose concentrations (*p* < 0.05), indicating that PEF-TPP displays a more pronounced in vitro hypoglycemic effect. Furthermore, it was observed that the GAC value of SDF samples displayed a significant positive linear correlation with their concentrations of glucose (*R*^2^ = 0.85, 0.94, and 0.94 for PEF40, PEF75, and PEF-TPP, respectively). Additionally, it was found that PEF-TPP exhibited the highest absorption rate compared to the other two methods (1.34 > 1.01 > 0.93). This enhanced adsorption capacity of PEF-TPP SDF can be attributed to its more porous network connection and larger surface area, which aligns with the findings of a previous study [36].

#### 2.3.2. Nitrite Ion Adsorption Capacity (NIAC)

The interaction between NO_2_^−^ and tertiary amines as well as secondary amines can lead to the conversion of nitrosamines in the stomach [4]. Additionally, it was indicated that NO_2_^−^ plays a significant role in scavenging toxic and harmful substances in the human body, particularly in the stomach, thereby providing a certain level of protection to human health [25].

According to the findings illustrated in Figure 1B, the scavenging capacity of NO_2_^−^ exhibited notable disparities between pH 2.0 and pH 7.0. This discrepancy implies that the scavenging efficacy of NO_2_^−^ primarily occurs within the gastric environment, effectively eliminating both endogenous and exogenous toxic agents to a certain degree, thereby safeguarding human well-being. The NIAC capabilities of the PEF-TPP technique exhibited a statistically significant increase compared to the PEF40 and PEF75 at a pH of 2.0 (*p* < 0.05). This phenomenon can be attributed to the specific surface characteristics resulting from separation, the development of a porous structure, and the notable reduction in particle size, consequently facilitating the enhanced absorption of hazardous nitrite ions [37].

#### 2.3.3. Cholesterol Adsorption Capacity (CAC)

The metabolism of cholesterol is intricately linked to the occurrence of cardiovascular and cerebrovascular diseases. In this context, it has been observed that SDF, by binding with food, can effectively diminish the digestion and absorption of cholesterol and triglycerides [36]. In this study, the simulated environments of the stomach and small intestine were set at a pH of 2.0 and a pH of 7.0, respectively. As illustrated in Figure 1C, the adsorption capacity of the SDFs was found to be higher at pH 7.0 compared to pH 2.0. The PEF-TPP method exhibited the highest cholesterol adsorption capacity among the SDF samples, while the PEF40 method showed the lowest adsorption capacity at both pH values. In comparison to the PEFs without TPP, the CAC values of PEF-TPP exhibited a significant increase within the time interval of 30–180 min (*p* < 0.05). Notably, there exists a strong positive linear relationship between the time and CAC at varying pH levels, while, the absorption characteristics showed some difference, as the biggest adsorption rate belonged to PEF-TPP and PEF75, respectively (2.58 > 1.83 > 1.49 at pH 2.0, and 3.20 > 2.67 > 1.65 at pH 7.0). The study revealed that PEF-TPP exhibited an elevated WHC and SC, while displaying a reduced presence of microcrystalline bundles. Upon water swelling, the PEF-TPP material underwent gelatinization, enabling it to effectively bind cholesterol within food, thereby mitigating its absorption. Additionally, in its amorphous state, the exposed active groups of PEF-TPP demonstrated a direct chelation of cholesterol molecules [38].

#### 2.3.4. Pancreatic Lipase Inhibition Capacity (PLIC)

Pancreatic lipase inhibition is a mechanism of lowering blood lipids [39]. Figure 1D illustrates that the inclusion of SDFs resulted in a noticeable reduction in lipase activity. Statistical analysis revealed a significant distinction between the PEF40, PEF75, and PEF-TPP SDFs (*p* < 0.05). Consequently, the PEF-TPP SDF exhibited the most potent inhibitory effect on pancreatic lipase activity, likely attributed to the higher concentration of enzyme-inhibiting groups in the fat-free composition of PEF-TPP SDF [40].

As previously outlined, the PEF-TPP SDF demonstrated notable efficacy in rapidly adsorbing glucose, nitrite ions, and cholesterol, as well as exhibiting superior pancreatic lipase inhibition capabilities.

#### 2.3.5. Antioxidant Capacities Analysis

The antioxidant properties of antioxidants have been associated with various mechanisms, including the inhibition of chain initiation, the binding of transition metal ion catalysts, the decomposition of peroxides, the prevention of ongoing hydrogen abstraction, the reductive capacity and the scavenging of radicals [29].

**Figure 2:**
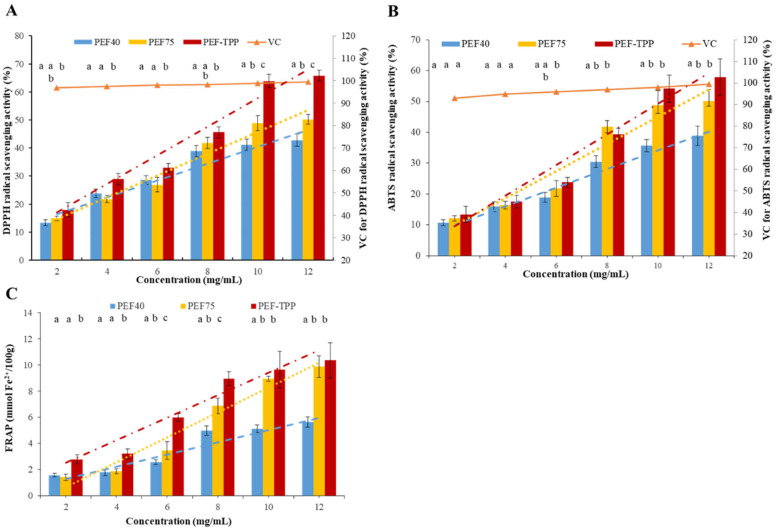
Antioxidant capacities of SDFs obtained via different extraction methods. DPPH radical-scavenging capacity (**A**); ABTS radical-scavenging capacity (**B**) and Ferric-reducing antioxidant power (**C**). Different letters are statistically different (*p* < 0.05).

Previous research has consistently demonstrated that the application of a PEF can enhance the antioxidant capacities of diverse bioactive extracts, including pigments [41], anthocyanins [42], and dietary fibers [35]. According to the data presented in Figure 2A–C, it can be observed that the DPPH and ABTS free radical-scavenging capacity, as well as the FRAP of the sample, exhibited a gradual increase with the escalating concentration of SDFs, ultimately reaching its peak value at 12 mg/mL.

The DPPH radical-scavenging assay is commonly used in antioxidant research to assess the ability of plant materials to provide hydrogen atoms and scavenge lipophilic free radicals [43]. In this study, the DPPH radical-scavenging activity of SDFs at various concentrations was examined, and it was found that the activity significantly increased (*p* < 0.05) as the concentration of SDFs increased from 2 to 10 mg/mL, as shown in Figure 2A. Notably, the DPPH radical-scavenging activity of SDFs obtained with PEF-TPP was found to be stronger compared to those obtained without TPP (*p* < 0.05). At a concentration of 10.0 mg/mL, the PEF-TPP demonstrated significantly higher amounts of scavenging activity on the DPPH radical (67.50%) compared to the PEF75 (48.10%) and PEF40 (41.50%). Furthermore, the scavenging capacities were further enhanced after the TPP method, as evidenced by the scavenging rates in the following order: PEF-TPP (10.21) > PEF75 (7.79) > PEF40 (5.99).

Furthermore, the IC_50_ value of PEF-TPP was found to be 4.24 mg/mL, demonstrating a lower value compared to that of PEF75 (5.54 mg/mL) and PEF40 (6.60 mg/mL). This suggests that PEF-TPP exhibits a superior scavenging capacity in comparison to other methods. It is important to note, however, that the scavenging capacity for the DPPH of the SDFs obtained via all three methods was weaker than that of the VC.

The ABTS assay is commonly used to evaluate the overall antioxidant capacity of individual compounds and diverse plant mixtures. In the range of low concentrations (2–4 mg/mL), the ABTS radical-scavenging ability of SDFs exhibited no statistically significant change (*p* > 0.05), as depicted in Figure 2B. However, a significant improvement (*p* < 0.05) in scavenging ability was observed with an increase in SDF concentration (4–10 mg/mL) for both the PEF75 and PEF-TPP, exhibiting similar scavenging rates. A similar value was reported for the SDF extracted via different methods from pear fruit pomace [18]. The IC_50_ values for the PEF75 (5.07 mg/mL) and PEF-TPP (5.56 mg/mL) were found to be similar, both of which were higher than the IC_50_ value for the PEF40 (7.61 mg/mL). Thus, the PEF-TPP SDF exhibited the highest scavenging capacity for ABTS, although it remained lower than that of the VC.

The FRAP method, known as the determination of total reductive power, was employed to assess a substance’s metal-reducing capacity in aqueous solutions. Antioxidant compounds facilitated the conversion of ferric (Fe^3+^) to its ferrous (Fe^2+^) form due to their inherent reductive potential [39]. According to the data presented in Figure 2C, it can be observed that the PEF-TPP SDF displayed notably higher absorbance, indicating that the PEF-TPP possessed a superior reducing capacity compared to that of the PEF75 and PEF40 (*p* < 0.05). It was widely anticipated that PEF-TPP SDF exhibited a certain degree of reducing power, potentially mitigating the occurrence of oxidative damage caused by free radicals in the human body.

The examination of the three distinct forms of antioxidant capacities revealed that the antioxidant capacity of SDF is contingent upon the concentration and adheres to the sequence of PEF-TPP > PEF75 > PEF40. The observed phenomenon can be attributed to the loose and porous structures, reduced molecular weight, and monosaccharide composition of PEF-TPP. These characteristics facilitate the donation of electrons or hydrogen atoms from hydroxyl groups in polysaccharides, resulting in the formation of stable free radicals and the termination of free radical reactions.

Yan proposed that antioxidant capacities are influenced by various factors, including the polymerization degree, monosaccharide composition, structure, molecular weight, and conformation of polysaccharides [29]. These characteristics facilitate the donation of electrons or hydrogen atoms from hydroxyl groups in polysaccharides, resulting in the formation of stable free radicals and the termination of free radical reactions. Hence, TPP treatment has the potential to enhance the antioxidant capacities of SDF, possibly as a result of the looser and more porous structures and reduced molecular weight.

### 2.4. Structural Analysis

#### 2.4.1. FT-IR Analysis

**Figure 3:**
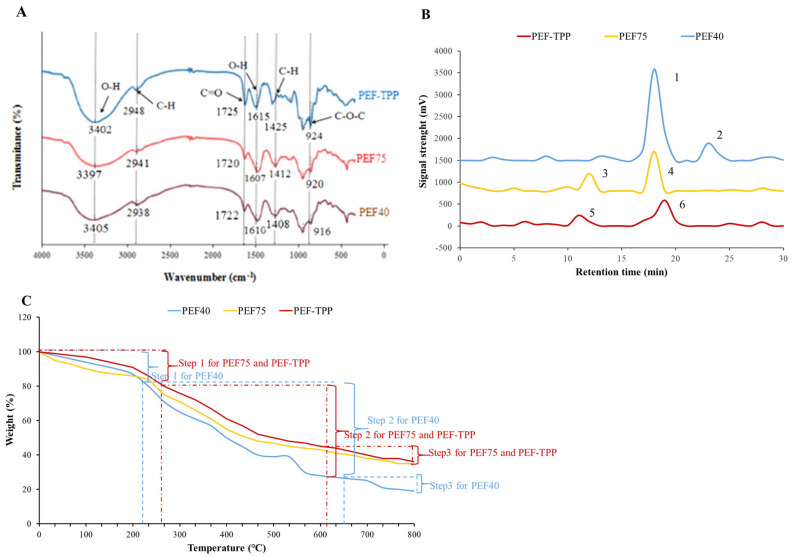
Structural characteristics of SDFs prepared using different extraction methods. FT-IR spectra of SDFs (**A**); HPGPC profiles of SDFs (**B**); TGA curves of SDFs (**C**).

FT-IR was utilized to examine the impact of various treatments on the chemical composition of SDF samples, as illustrated in Figure 3A. With the exception of a few distinctive bands, all SDFs exhibited comparable spectral profiles pertaining to their primary constituents and overall chemical structure. Each sample displayed prominent and broad absorption peaks at approximately 3400 cm^−1^, indicative of the hydrogen bonding involving polysaccharides and the stretching of O-H bonds [44]. The peak observed at approximately 2940 cm^−1^ corresponds to the vibrational stretching of the C-H bond in either methyl or methylene groups, as reported by the previous report [45]. Additionally, the absorption peaks detected at 1722 cm^−1^ and 1610 cm^−1^ are indicative of the presence of C=O and O-H bonds, respectively, suggesting the existence of aldehyde acids within the SDF sample. Furthermore, the peak observed at 1410 cm^−1^ signifies the bending vibration of the C-H bond, while the narrow and intense peak at 920 cm^−1^ can be attributed to the stretching vibration of the C-O-C bond in the glucopyranoside [46]. The PEF-TPP SDF exhibited the most substantial O-H peak area, suggesting a greater presence of intramolecular hydrogen bonds and, consequently, a heightened degree of hydrophilicity.

#### 2.4.2. Mw Distribution Analysis

The molecular weights (Mw, Mn, and Pd) of the three SDFs derived from peanut shells were determined using HPGPC. The findings are displayed in Table 3 and Figure 3B. Notably, the distinct extraction methods yielded substantial variations in the molecular weights of the SDFs. Previous studies have indicated that diverse extraction methods have the potential to generate dissimilar molecular weights, thereby influencing the functional activity of SDF [47]. The PEF40, PEF75, and PEF-TPP exhibited bimodal distributions, with the primary peak displaying molecular weights of 324 kDa, 268 kDa, and 245 kDa, and retention times of 18.25 min, 12.31 min, and 11.29 min, respectively (Table 3). In comparison to the PEF40 and PEF75, the PEF-TPP SDF exhibited the lowest molecular weight. This occurrence can be attributed to the degradation of the cellulose and hemicellulose molecular chains induced by high voltage, resulting in the reduced molecular polymerization and heightened solubility of the PEF-TPP SDF [48]. According to Silva [49], prior research has demonstrated that low molecular weight DF is more effective in reducing cholesterol absorption and facilitating its excretion in feces. A lower polydispersity (Pd) coefficient typically signifies a more concentrated and uniform molecular weight distribution. The presence of a relatively low Pd value (Mw/Mn = 2.58) in Peak 5 of the PEF-TPP SDF suggests that it possesses a concentrated and uniform distribution of molecular weight.

#### 2.4.3. Monosaccharide Composition Analysis

The monosaccharide compositions of the SDF samples obtained from peanut shells using various extraction methods were examined. Table 4 presents a summary of the predominant monosaccharides found in the SDF samples, including rhamnose, arabinose, galactose, glucose, xylose, mannose, fructose, and galacturonic acid. Arabinose emerged as the predominant monosaccharide, succeeded by galacturonic acid and galactose. Nevertheless, rhamnose, xylose, and galacturonic acid were absent in the alkaline hydrogen peroxide (AHP) modification. This phenomenon could potentially be attributed to the AHP’s disruption of glycosidic bonds, leading to the formation of novel monosaccharides within the SDF samples [50].

In comparison to the PEF40 and PEF75, there was a significant increase in the levels of arabinose, galactose, glucose, mannose, and fructose in the PEF-TPP SDF. This suggests that a substantial portion of the cellulose has undergone degradation, and a fraction of the hemicellulose has been liberated [21]. There was speculation regarding the potential impact of the PEF-TPP treatment on the molecular structure, specifically in terms of the extent of branching and linearity exhibited by the main chains [36].

Table 4 presents the findings that the PEF-TPP SDF exhibited a substantial presence of neutral sugars, along with a higher proportion of the RG-I region (2 Rha + Ara + Gal) compared to the HG region (GalA-Rha). These results suggest a lower level of linearity, increased branching in the RG-I region, and a greater degree of branching overall. Consequently, the sample displayed a higher number of bondage chains and intermolecular forces, such as hydrogen bonding and hydrophobic interactions. These characteristics contribute to the enhanced viscosity and viscoelasticity [51,52,53].

#### 2.4.4. TG Analysis

The thermal stability of different SDFs was evaluated by obtaining the weight loss and thermal degradation temperature characteristics during temperature changes using a TGA. The thermogravimetric (TG) curves of the various SDFs can be observed in Figure 3C. The thermal decomposition the SDFs obtained using the PEF40, PEF75, and PEF-TPP exhibited a three-step process. The initial stage of thermal decomposition for the untreated sample occurred between 30–240 °C, primarily due to the evaporation of free water and crystal-bound water [54]. Consequently, the sample weights were reduced by 14.82%. This phenomenon can be attributed to the augmented presence of shorter chains in the PEF-TPP SDF and the reinforced hydrogen bonding at binding sites, necessitating a greater amount of energy for structural disruption [55]. In all instances, the peak weight reduction was observed during the second stage, which corresponds to the degradation of pectin, hemicellulose, and other saccharides. This phenomenon can be attributed to the augmented presence of shorter chains in the PEF-TPP SDF and the reinforced hydrogen bonding at binding sites, necessitating a greater amount of energy for structural disruption [50]. In all instances, the peak weight reduction was observed during the second stage, which corresponds to the degradation of pectin, hemicellulose, and other saccharides. The second stage’s temperature range for the PEF40 was observed to be between 220 and 650 °C, with corresponding mass loss rates of 61.46%. In the case of the PEF75 and PEF-TPP, the second stage’s temperature range was found to be between 260 and 610 °C, resulting in mass loss rates of 36.03% and 35.84%, respectively. During the final phase, the rate of mass loss exhibited a gradual and consistent pattern. Specifically, the mass-loss rates for the PEF40, PEF75, and PEF-TPP SDFs were determined to be 2.64%, 4.59%, and 6.84%, respectively. Similarly, SDF derived from Rubus chingii Hu. Fruits, defatted coconut flour, lotus root, and lemon peel displayed a comparable three-step thermal decomposition process, with the highest weight reduction occurring in the second stage [45,46,56,57]. Furthermore, in the cases of three peanut shell samples, it was observed that the residual qualities of the PEF75 and PEF-TPP SDFs were comparatively elevated, suggesting that the thermal properties of the SDFs were impacted by the PEF extraction techniques. Additionally, it was found that the SDF obtained through the PEF-TPP extraction exhibited superior thermal stability.

#### 2.4.5. SEM Analysis

**Figure 4:**
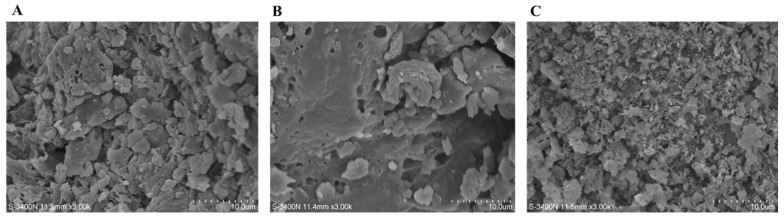
The SEM of SDFs prepared using PEF 40 (**A**), PEF 75 (**B**) and PEF-TPP (**C**).

Figure 4 depicts the scanning electron microscope (SEM) images of the SDFs through various extraction techniques. The SEM analysis reveals notable distinctions in the morphologies of the PEF40, PEF75, and PEF-TPP SDFs. Specifically, the untreated sample exhibits a dense structure characterized by a wrinkled surface with visible cracks and holes (Figure 4A). Conversely, the surfaces of the PEF75 and PEF-TPP SDFs appear to be less compact and more porous compared to those of the untreated sample (Figure 4B,C). In contrast to the PEF75, the PEF-TPP exhibited enhanced pore uniformity and a more relaxed spatial arrangement, leading to a substantial augmentation in surface area, a facilitation of water absorption and expansion, and an enhancement in the SDF’s capacity to absorb glucose, cholesterol, and detrimental substances within the gastrointestinal tract [13]. Comparable findings were observed when employing pulsed electric fields to modify dietary fiber derived from orange peels [37].

#### 2.4.6. Correlation between Antioxidant Capacities and Structures of the SDFs

**Figure 5:**
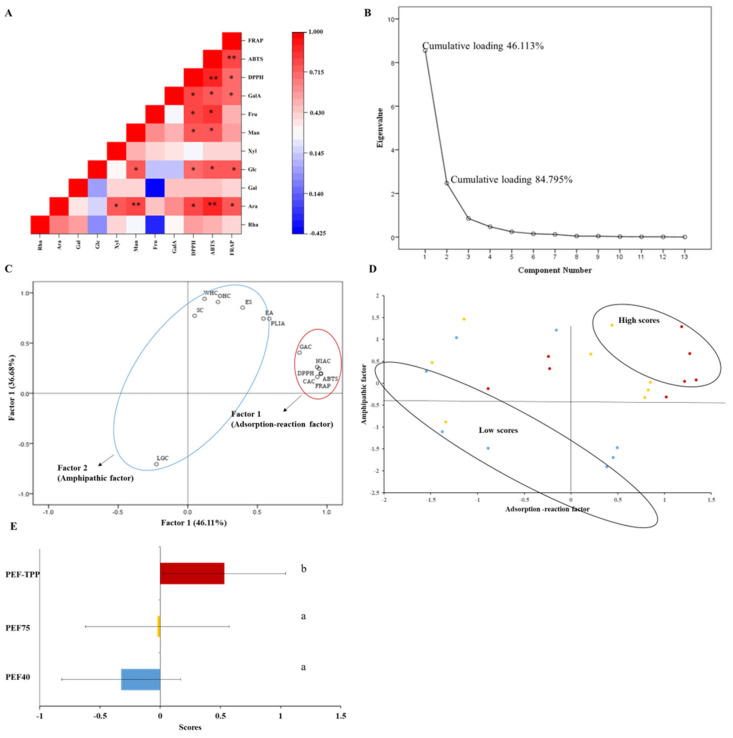
The evaluation of the characteristics of the SDFs obtained using different methods. (**A**) Heat map of Pearson’s correlation coefficient among the antioxidant capacities and monosaccharide components. Gradient color barcodes at the right indicate the minimum value in blue and the maximum in red (for the interpretation of the references to the colors in this figure’s legend). * Significant at *p* < 0.05, ** Significant at *p* < 0.01; (**B**) Screen plot of the PCA of the characteristics of SDFs obtained using different methods; (**C**) The loading scatter plot of the PCA of the characteristics of SDFs obtained using different methods; (**D**) PCA score scatter plot of characteristics of SDFs obtained using different methods (red spots are PEF-TPP scores, yellow spots are PEF75 scores, and blue spots are PEF40 scores); (**E**) The synthesis scores of the three methods tested.

The Pearson correlation coefficient was employed to ascertain the presence of a correlation between the primary monosaccharide constituents and their corresponding antioxidant capacity (Figure 5A). The DPPH radical-scavenging capacity was highly correlated with Ara (*r* = 0.81, *p* < 0.05), Glc (*r* = 0.70, *p* < 0.05), Man (*r* = 0.75, *p* < 0.05), Fru (*r* = 0.77, *p* < 0.05) and GalA (*r* = 0.78, *p* < 0.05). Meanwhile, the ABTS radical-scavenging capacity exhibited comparable correlations. Furthermore, the FRAP were significantly positively correlated with Ara (*r* = 0.75, *p* < 0.05), Glc (*r* = 0.75, *p* < 0.05), and GalA (*r* = 0.70, *p* < 0.05). It is plausible to hypothesize that the increased antioxidant capacities of PEF-TPP may be attributed, at least in part, to its higher levels of Ara, Glc, and GalA, which aligns with findings from a prior study [58].

### 2.5. Principal Component Analysis

In order to evaluate the most effective approach for obtaining SDF, a principal component analysis (PCA) was employed utilizing various characteristic values. Generally, KMO values exceeding 0.50 and *p* values of 0.05 for Bartlett’s sphericity test are deemed acceptable. The PCA conducted in this study unveiled that multiple factors play a role in determining the quality of the SDF. Figure 5B illustrates the existence of two components possessing eigenvalues exceeding 1, which collectively contribute to 84.795% of the SDF’s quality. In accordance with the statistical criteria presented in Figure 4C, the adsorption-reaction factor (component 1) encompasses the DPPH, ABTS, FRAP, GAC, CAC, and NIAC, while the amphipathic factor (component 2) encompasses the LGC, ES, EA, PLIA, SC, WHC, and OHC. These aforementioned factors effectively account for the characteristics of the SDFs.

Based on the variance contribution of the two factors as the weight, a comprehensive evaluation model was established to determine the quality of the SDFs. The model, represented by F = (0.46F_1_ + 0.39F_2_)/0.848, uses the value of F as a score to reflect the quality. The analysis of Figure 5D revealed that the top five high scores consisted of four from the PEF-TPP method and one from the PEF75 method, while the top five low scores consisted of four from the PEF40 method and one from the PEF75 method. Consequently, the highest score among the SDFs was obtained using the PEF-TPP method, which was significantly higher than those obtained using PEF40 and PEF75 (Figure 5E).

As previously discussed, treatment with TPP has been shown to enhance various processing and functional properties of SDF, including the WHC, OHC, SC, EA, ES, and LGC. These improvements can be attributed to the unique structure of SDF, characterized by a loose and porous surface, small molecular mass, and a high content of arabinose, galactose acid, glucose, mannose, and fructose with extensive branching. The modification in the SDF structure may be attributed to the application of the PEF and TPP treatment.

## 3. Materials and Methods

### 3.1. Materials and Reagents

The peanut shells (PSs) were provided by Tangshan Runze Cereals, Oils and Food Co., Ltd. (Tangshan, China). The PS sample underwent a drying procedure at −80 °C in a vacuum freeze dryer for a duration of 24 h. Following this, the sample was pulverized utilizing an XA-I high-speed pulverizer, and sieved through an 80-mesh screen. Subsequently, the resulting powder was transferred to a dry container for further analysis.

The standard monosaccharides were procured from Sigma-Aldrich (St. Louis, MO, USA). All chemicals and reagents utilized in this study were of an analytical grade and were obtained from Jiangtian Chemical Technology Co. Ltd. (Tianjin, China).

The study employed the following instruments: the XD 5000 rotary evaporator, GL-20G-Ⅱ high-speed refrigerated centrifuge, and HH-4 thermostatic water bath, as well as EX-1900 pulsed electric fields equipment (manufactured by Xinan technology company, Guangzhou, China).

### 3.2. Preparation of SDF

**Figure 6:**
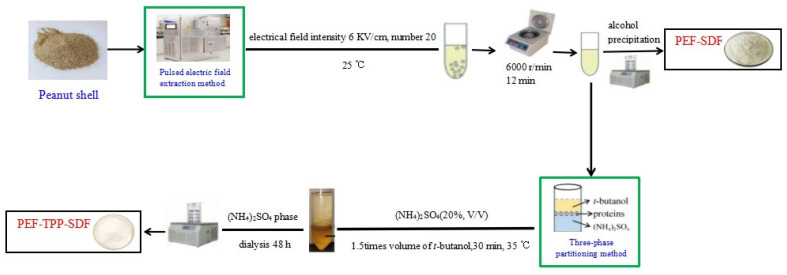
Schematic procedures for the preparation of dietary fiber from peanut shells using pulsed electric fields with (and without) three-phase partitioning.

The specific preparation processes are presented in Figure 6. A pulsed electric field was used to assist in the extraction of the PEF. There are three methods adopted to separate the SDF. The traditional method consisted of ethanol precipitation, and the high efficiency separation method consisted of three-phase partitioning.

#### 3.2.1. Extraction of SDF

In accordance with prior research, the PEF parameters were established as follows: an electrical field intensity of 8 kV/cm, a fixed number of 20, a frequency of 1 Hz, and a pulse width of 20 μs [15].

#### 3.2.2. Separation of SDF

##### Ethanol Precipitation

The supernatant obtained through PEF extraction was precipitated overnight with four times the volume of ethanol with different concentrations (40% or 75%). The resulting sediment was thoroughly dried to yield the SDF, denoted as PEF40 and PEF75, respectively.

##### Three-Phase Partitioning

First, PEF was performed, remaining consistent with the aforementioned parameters. Then, the supernatant obtained through PEF extraction was concentrated to 50% of its initial volume and subsequently combined with 20% (*w*/*w*) (NH_4_)_2_SO_4_ while being continuously stirred. Following the completed mixing, a 1.5-fold volume of tert-butanol was introduced into the mixture. The resulting mixture was then subjected to continuous agitation using an electronic stirrer for a duration of 30 min at a temperature of 35 °C. There are three phases in the separation process. The upper phase consists of tert-butanol, the middle phase contains some residue, and the lower phase includes SDF and (NH_4_)_2_SO_4_. After centrifugation at 8000 r/min for 5 min, the lower phase containing (NH_4_)_2_SO_4_ was isolated and subjected to dialysis using a 3500 Da dialysis bag for a period of 48 h. The (NH_4_)_2_SO_4_ diffused out of the dialysis tube, and BaCl_2_ was introduced to the dialysate. The completion of dialysis was confirmed by the absence of a white precipitate. Subsequently, the sample was concentrated and lyophilized to yield the SDF, referred to as the PEF-TPP SDF.

### 3.3. Chemical Composition Analysis

The total sugar content was analyzed using the AOAC 968.28 method [59], while the dietary fiber content was determined using the AOAC 991.43 method [60].

### 3.4. Physicochemical Properties

#### 3.4.1. Water-Holding Capacity (WHC), Oil-Holding Capacity (OHC), and Swelling Capacity (SC)

The water-holding capacity (WHC) of the SDF was determined using Wang’s method [36]. In this procedure, 0.5 g of the SDF was combined with 5.0 mL of distilled water and agitated for 30 s. The resulting mixture was then allowed to settle for 24 h at a temperature of 25 °C. Subsequently, the mixture was subjected to centrifugation at a speed of 4200 rpm for a duration of 15 min. The sediment obtained from this process was collected and weighed in order to calculate the WHC using Equation (1):

(1) $$\mathrm{WHC}\;(g/g)=\frac{W_{1}-W_{0}}{W}$$

The variables are denoted as follows: W_1_ represents the combined weight (in grams) of the centrifuge tube prior to the centrifugation process. W_0_ represents the combined weight (in grams) of the centrifuge tube and only the sediment. Lastly, W represents the weight (in grams) of the SDF sample.

The oil-holding capacity (OHC) of the SDF was determined using a modified version of the method described by Wang [36]. In this procedure, 0.5 g of the SDF was combined with 5.0 mL of olive oil, and the mixture was suspended for 30 s at a temperature of 25 °C for a duration of 6 h. Subsequently, the mixture was subjected to centrifugation at 7000 rpm for 15 min, resulting in the removal of the supernatant. The remaining sediment was then weighed, and the OHC was calculated using Equation (2).

(2) $$\mathrm{OHC}\;(g/g)=\frac{W_{1}-W_{0}}{W}$$

The variables are denoted as follows: W_1_ represents the combined weight (in grams) of the centrifuge tube prior to the centrifugation process. W_0_ represents the combined weight (in grams) of the centrifuge tube and only the sediment. Lastly, W represents the weight (in grams) of the SDF sample.

The swelling capacity (SC) was assessed using the methodology described by Zhang [61]. A tube containing 0.2 g of dry SDF and 5 mL of water was subjected to hydration for a duration of 18 h at a temperature of 4 °C. The resulting volume occupied by the SDF was measured in order to compute the SC, employing Equation (3).

(3) $$\mathrm{SC}\;(\mathrm{mL}/g)=\frac{V}{W}$$

In this context, V denotes the final volume of the SDF sample, while W represents the SDF sample itself.

#### 3.4.2. Emulsifying Activity, Emulsion Stability, and Least Gelation Concentration

The estimation of the emulsifying activity (EA) and emulsion stability (ES) was conducted using Chao’s method with slight adjustments [62]. Specifically, 2 g of SDF was dispersed in 100 mL of deionized water and homogenized at a speed of 2000 rpm for a duration of 2 min. Subsequently, 100 mL of corn oil was introduced and homogenized for 1 min to achieve the emulsion. This emulsion was then subjected to centrifugation at 1200 rpm for 5 min, followed by a measurement of the emulsion volume. The EA was determined by employing Equation (4):

(4) $$\mathrm{EA}\;(\mathrm{mL}/100\mathrm{mL})=\frac{V_{1}}{V}\times 100$$

where V_1_ and V represent the volumes of the emulsified layer and the total liquid, respectively.

The emulsion underwent a heating process at a temperature of 80 °C for a duration of 30 min, followed by a cooling process to reach a temperature of 25 °C. Subsequently, it was subjected to centrifugation at a speed of 1200 rpm for a duration of 5 min. The calculation of the ES was performed utilizing Equation (5):

(5) $$\mathrm{ES}\;(\mathrm{mL}/100\mathrm{mL})=\frac{V_{1}}{V}\times 100$$

where V_1_ and V are the volumes of the emulsified layer after and before heating, respectively.

The determination of the least gelation concentration (LGC) was carried out according to a previously published method with some adjustments [63]. SDF powder was dissolved in distilled water at concentrations ranging from 2% to 12% (*w*/*v*) to prepare the suspension. Following this, the suspensions (5 mL) underwent a heat treatment at 100 °C for 1 h, followed by cooling in an ice bath for 1 h. The LGC was determined by identifying the point at which the suspensions remained solidified even after inversion and vigorous shaking.

### 3.5. Functional Properties

#### 3.5.1. Glucose Adsorption Capacity (GAC)

The GAC was determined using the methodology described by Zheng [64]. Specifically, 0.5 g of each sample was combined with 50 mL of a glucose solution (50–100 mmol/L). The resulting mixture was subjected to agitation at a temperature of 37 ℃ for a duration of 6 h, followed by centrifugation at a force of 5000× *g* for a period of 12 min. Subsequently, the content of the reducing sugar in the supernatant was measured. The GAC was then calculated utilizing Equation (6):

(6) $$GAC\;(\mathrm{mmol}/g)=\frac{\left(A_{i}-A\right)\times v}{m}$$

where A_i_ represents the reduced sugar content in the blank group, in g/100 g, and A represents the reducing sugar content in the experimental group, in g/100 g; v denotes the volume of the solution, and m signifies the weight of the sample in grams.

#### 3.5.2. Pancreatic Lipase Inhibition Capacity (PLIC)

The PLIC was evaluated using the methodology described by Chau [65], which involved combining 0.5 g of each sample with 1 mL of pancreatic lipase solution (0.2 mg/mL), 10 mL of soybean oil, and 50 mL of a phosphate buffer (0.1 mol/L, pH = 7.2). The resulting mixture was then stirred magnetically at 37 °C for 1 h and subsequently cooled in an ice bath for 10 min to stop the reaction. Following this, 2 drops of phenolphthalein indicator (10 g/L) were added, and the solution was titrated with 0.1 mol/L of a NaOH solution to quantify the amount of fatty acid released. The assessment of lipase activity inhibition involved comparing the rate of fatty acid reduction to that of the control. The PLIC was determined using Equation (7):

(7) $$\mathrm{PLAI}\;\mathrm{Inhibition}\;(\%)=\frac{\left(V-V_{1}\right)\times C\times M}{V\times C\times M}$$

where V is the volume of NaOH consumed in the absence of the sample, in mL; C is the concentration of NaOH in the standard solution used for titration, in mL; V_1_ is the volume of NaOH consumed during titration, in mL; M is the molar mass of the free fatty acids, in g/moL.

#### 3.5.3. Cholesterol Adsorption Capacity (CAC)

The determination of the CAC was conducted using the experimental methodology outlined by Jia [66]. In brief, a homogeneous emulsion was prepared by beating yolks and deionized water in a ratio of 1:9 (*v*/*v*). Subsequently, 1 g of the sample was combined with 20 mL of diluted yolk. The pH was adjusted to either 2.0 or 7.0, and the mixture was incubated in a shaker water bath at 37 °C for varying durations of 30, 60, 90, and 180 min. Following incubation, the mixture was subjected to centrifugation at 5000× *g* for 18 min.

The cholesterol concentration in the resultant supernatant was quantified through the o-phthalaldehyde method, and the CAC was computed using Equation (8).

(8) $$CAC\;(mg/g)=\frac{\left(C_{y}-C_{d}\right)-(C_{y}-C_{b})}{W}\times 20$$

where W is the weight of the SDFs, C_y_ is the diluted yolk, as a negative group, and C_b_, C_d_ are the cholesterol concentration in the diluted yolk without SDFs and with SDFs, respectively; 20 is the adsorption volume, in mL.

#### 3.5.4. Nitrite Ion Adsorption Capacity (NIAC)

The detection of the NIAC followed the methodology outlined by Zhu [67]. Each sample, weighing 0.1 g, was combined with 5 mL of a NaNO_2_ solution (20 g/mL). The pH was adjusted to either 2.0 or 7.0 to replicate the conditions of the stomach and small intestine.

The resulting mixture was subjected to agitation at a temperature of 37 °C for a duration of 2.5 h, followed by centrifugation at a force of 4500× *g* for 12 min. Subsequently, 0.3 mL of the resulting supernatant was transferred to a tube, and 1.7 mL of deionized water was added. Following this, solutions of p-aminobenzene sulfonic acid (2 mL, 4 g/mL) and naphthalene diamide hydrochloride (1 mL, 2 g/mL) were introduced. The concentration of the NaNO_2_ was then determined at a wavelength of 538 nm and quantified using the established standard curve. The NIAC was calculated utilizing Equation (9).

(9) $$NIAC\;(µg/g)=\frac{\left(m-m_{1}\right)}{W}$$

m and m_1_ represent the weights of the NaNO_2_ before and after adsorption, respectively, with W being the weight of the SDF sample.

#### 3.5.5. Antioxidant Capacity

The DPPH radical-scavenging capacity of the samples was assessed using the method described by Wang [36], with minor modifications. A solution of DPPH in methanol, with a concentration of 6 × 10^−5^ mol/L, was prepared. Subsequently, 3 mL of the DPPH solution was combined with 100 μL of the SDF solution, which had been diluted. The resulting mixture was incubated in darkness at 37 °C for 20 min, after which the absorbance decrease caused by the addition of the antioxidants (SDF) was measured at a wavelength of 515 nm. This experiment was replicated three times, and the VC (0.01 mg/mL) was selected as the positive control.

The compound 2,2′-azino-*bis*(3-ethylbenzothiazoline-6-sulfonic acid) (ABTS) was dissolved in deionized water to create a stock solution with a concentration of 7 mmol/L. A working solution of ABTS was prepared by combining it with 2.45 mM of potassium persulfate and allowing it to incubate in darkness at a temperature of 25 °C for a duration of 12–16 h. The ABTS^·+^ solution was then diluted in deionized water until it reached an absorbance of 0.7 (±0.02) at a wavelength of 734 nm. Subsequently, a 0.1 mL sample (appropriately diluted) was mixed with 3 mL of the ABTS^·+^ working solution and allowed to react in darkness for a period of 30 min. The experiment was repeated three times, and the VC (0.01 mg/mL) was selected as the positive control.

Free radical-scavenging activity was calculated using the following formula:

(10) $$scavengingactivity\;(\%)=\frac{\left(A_{C}-A_{E}\right)}{A_{C}}\times 100$$

where A_c_ and A_E_ relate to the absorbance of DPPH/ABTS without or with SDF, respectively.

The ferric ion-reducing antioxidant power (FRAP) was assessed following the methodology outlined by Wang [36].

We prepared a FRAP solution by mixing 10 mL of an acetate buffer (300 mmol/L, pH 3.6) and 1 mL of TPTZ (10 mmol/L) with an HCl solution of 40 mmol/L, and then adding 20 mmol of ferric chloride. Afterwards, 1 µL of the sample solution was mixed with 300 μL of deionized water and diluted with 3 mL of the freshly prepared FRAP solution. Using the method outlined above, the resulting mixture was incubated at 37 °C for 30 min and measured at 593 nm. The dose–response curves of FeSO_4_·7H_2_O were calculated. The results were expressed as an FeSO_4_ equivalent.

### 3.6. Structural Analysis

#### 3.6.1. Fourier-Transform Infrared (FT-IR) Spectra Analysis

The FT-IR spectra of the SDF samples were measured using a Thermo Scientific Nicolet iS50 FT-IR spectrometer (Shanghai, China). Prior to FT-IR scanning in the frequency range of 4000–400 cm^−1^, the dried SDF samples were ground with potassium bromide (KBr) powder and then pressed into pellets (2 mg SDF sample/200 mg KBr).

#### 3.6.2. Molecular Weight (Mw) Distribution

In a prior investigation carried out by Wang [36], the molecular weight of SDF was ascertained utilizing high performance liquid chromatography with a refractive index detector (RI-10A; Shimadzu Corp., Tokyo, Japan) and a BRT105-104-102 column (8 mm × 300 mm). The mobile phase utilized in the experiment comprised a 0.05 M NaCl solution and was eluted at a flow rate of 0.6 mL/min over a period of 60 min. The column temperature was held constant at 40 °C, with an injection volume of 20 µL. The SDF sample was prepared in an aqueous solution at a concentration of 5 mg/mL and subsequently filtered through a 0.22 µm filter. A standard dextran curve was generated to determine the molecular weight of the SDFs.

#### 3.6.3. Monosaccharide Composition

The monosaccharide composition of the samples was determined using HPLC according to the method described by Yuan [68]. Briefly, 0.01 g of the SDF sample was mixed with 2 mL of 2 mol/L trifluoroacetic acid and hydrolyzed at 100 °C for 8 h. The resulting mixture was then dried, washed with 1 mL of methanol, and dissolved in 1 mL of distilled water.

Subsequently, the hydrolyzed SDF was subjected to derivatization using a 0.5 mol/L PMP-methanol solution and 0.3 mol/L of NaOH for a duration of 1 h at a temperature of 70 °C. Following the cooling of the reaction mixture to ambient temperature, the resulting product was neutralized with 300 µL of 0.3 mol/L of HCl and 1 mL of chloroform. Finally, the mixture was centrifuged at 4800 rpm for 10 min. After the absorption of the supernatant, 1 mL of chloroform was subsequently introduced for three consecutive extractions, with the final supernatant being filtered through a 0.22 µm membrane. The injection volume was set at 20 µL. Acetonitrile and a 0.1 mol/L phosphate buffer with a pH of 6.7 were employed as mobile phases A and B, respectively, in an 18:82 ratio. The flow rate was maintained at 1 mL/min.

#### 3.6.4. Thermogravimetric (TG) Analysis

The thermal properties analysis was conducted on 10 mg samples of SDF utilizing TGA/DSC within the temperature range of 30 to 300 °C. The heating rate employed was 5 °C/min, accompanied by a liquid nitrogen flow rate of 50 mL/min. For the determination of particle diameter and specific surface analysis, a laser particle analyzer (BT-9300H) was employed.

#### 3.6.5. Surface Morphological Analysis

The scanning electron images (SEM) of the SDFs subjected to various treatments were acquired using a scanning electron microscope (JSM-6360LV, JEOL, Tokyo, Japan).

The specimens were mounted onto a specimen holder using double-sided scotch tape and then coated with a layer of gold through sputter-coating (10 min, 2 millibars). Following this, the samples were transferred to the scanning electron microscope for observation at a magnification of 3000× with an accelerating voltage of 15.0 kilovolts.

### 3.7. Statistical Analysis

The statistical software utilized in this study was SPSS 20.0 (SPSS Inc., Chicago, IL, USA). All results were presented as means ± standard deviation (S.D.). The data underwent an analysis of variance (ANOVA), and significant differences (*p* < 0.05) among means were assessed using Duncan’s multiple-range test. Principal component analysis (PCA) was performed using SPSS Statistics 20. Initial data analysis and plotting were conducted using Origin Pro 2020. Statistical significance was determined with a *p*-value of less than 0.05 indicating significant differences.

## 4. Conclusions

In this study, the PEF extraction method, in conjunction with TPP, was employed to extract the SDF from PSs. The subsequent analysis involved the examination of its chemical composition, structural characterization, physicochemical attributes, and functional properties, with a comparative assessment against the absence of TPP utilization. The results of the study suggest that treatment with TPP may improve the thermal stability and decrease the molecular weight, potentially enhancing the functional and processing properties of the material. Furthermore, the composition of the PEF-TPP SDF is more complex, leading to superior performance compared to the SDF prepared using other methods, as indicated by the higher score achieved. This suggests that the PEF-TPP extraction technique holds significant promise for the production of SDFs in the functional food industry.

## Figures and Tables

**Table 1:** Proximate composition of the PS, SDFs obtained with different methods.

Proximate Composition (g/100 g)	PS		Obtained SDFs	
		PEF40	PEF75	PEF-TPP
Dietary fiber	83.91 ± 0.75 ^a^	86.04 ± 0.53 ^b^	90.04 ± 0.43 ^c^	96.02 ± 0.58 ^d^
Protein	5.89 ± 0.47 ^a^	4.48 ± 0.22 ^b^	3.33 ± 0.28 ^c^	0.87 ± 0.14 ^d^
Fat	4.45 ± 0.32 ^a^	2.08 ± 0.21 ^a^	1.68 ± 0.11 ^b^	1.33 ± 0.07 ^c^
Moisture	2.84 ± 0.11 ^a^	1.93 ± 0.12 ^b^	1.71 ± 0.08 ^c^	1.01 ± 0.05 ^d^
Ash	1.78 ± 0.04 ^a^	1.51 ± 0.11 ^b^	1.21 ± 0.04 ^c^	0.95 ± 0.12 ^d^
Total sugar	11.25 ± 0.25 ^a^	22.47 ± 0.62 ^b^	31.47 ± 1.47 ^c^	33.18 ± 1.27 ^c^
Yield (%)	-	27.31 ± 1.42 ^a^	22.31 ± 2.12 ^b^	21.43 ± 2.45 ^b^

The values represent means of triplicates ± standard deviation. Values in the same row with different letters are significantly different (*p* < 0.05).

**Table 2:** Physicochemical properties of SDFs which underwent treatment with PEF40, PEF75, and PEF-TPP.

Samples	WHC (g/g)	OHC (g/g)	SC (mL/g)	EA (mL/100 mL)	ES (mL/100 mL)	LGC (%)
PEF40	3.98 ± 0.29 ^a^	2.88 ± 0.20 ^a^	4.57 ± 0.36 ^a^	66.37 ± 1.83 ^a^	55.11 ± 1.13 ^a^	11.26 ± 0.71 ^a^
PEF75	4.96 ± 0.17 ^b^	3.74 ± 0.14 ^b^	5.49 ± 0.27 ^b^	73.69 ± 1.01 ^b^	63.54 ± 1.24 ^b^	10.02 ± 0.31 ^b^
PEF-TPP	5.67 ± 0.67 ^c^	3.89 ± 0.41 ^b^	6.96 ± 0.88 ^c^	79.69 ± 2.36 ^c^	70.36 ± 2.13 ^c^	8.18 ± 0.28 ^c^

The values represent means of triplicates ± standard deviation. Values in the same column with different letters are significantly different (*p* < 0.05). (WHC: water-holding capacity; OHC: oil-holding capacity; SC: swelling capacity; EA: emulsifying activity; ES: emulsion stability; LGC: least gelation concentration).

**Table 3:** Effects on molecular weight of SDFs extracted using PEF40, PEF75 and PEF-TPP.

Sample	Peak Number	RT (min)	Mw (kDa)	Mn (kDa)	Pd (Mw/Mn)
PEF40	1	18.25	324	104	3.12
	2	23.48	142	78	1.82
PEF75	3	12.31	268	81	3.31
	4	18.01	115	52	2.21
PEF-TPP	5	11.29	245	95	2.58
	6	18.95	110	47	2.34

Values are given as means of independent experiments. Weight-average molecular weight (Mw); Number-average molecular weight (Mn); Polydispersity (Pd).

**Table 4:** Monosaccharide composition of SDFs extracted using PEF40, PEF75 and PEF-TPP.

Monosaccharide (mg/g db)	PEF40	PEF75	PEF-TPP
Rhamnose (Rha)	11.68 ± 1.14	12.05 ± 0.95	12.47 ± 1.24
Arabinose (Ara)	68.54 ± 3.58	72.61 ± 2.58	78.32 ± 5.21
Galactose (Gal)	18.98 ± 2.01	19.05 ± 1.35	20.13 ± 1.34
Glucose (Glc)	15.12 ± 1.45	15.21 ± 1.05	21.17 ± 0.64
Xylose (Xyl)	15.60 ± 1.51	16.98 ± 1.75	16.48 ± 1.56
Mannose (Man)	10.57 ± 1.37	13.25 ± 1.21	15.52 ± 0.54
Fructose (Fru)	6.02 ± 1.06	7.48 ± 1.35	7.32 ± 0.54
Galacturonic acid (GalA)	33.25 ± 3.44	36.98 ± 2.18	35.48 ± 2.54
HG = GalA − Rha	21.59 ± 1.86	24.93 ± 2.14	21.86 ± 3.04
RG-I = 2 Rha + Ara + Gal	110.88 ± 2.15	115.84 ± 1.95	123.39 ± 13.10

## Data Availability

The data is unavailable due to privacy or ethical restrictions.
